# Population uptake of antiretroviral treatment through primary care in rural South Africa

**DOI:** 10.1186/1471-2458-10-585

**Published:** 2010-09-29

**Authors:** Graham S Cooke, Frank C Tanser, Till W Bärnighausen, Marie-Louise Newell

**Affiliations:** 1 Africa Centre for Health and Population Studies, University of KwaZulu-Natal, PO 198, Mtubatuba 3935, KwaZulu-Natal, South Africa; 2 Division of Infectious Diseases, Imperial College, London W2 1NY, UK; 3 Department of Global Health and Population, Harvard School of Public Health, Boston, MA, USA; 4 UCL Institute of Child Health, University College London, London, UK

## Abstract

**Background:**

KwaZulu-Natal is the South African province worst affected by HIV and the focus of early modeling studies investigating strategies of antiretroviral treatment (ART) delivery. The reality of antiretroviral roll-out through primary care has differed from that anticipated and real world data are needed to inform the planning of further scaling up of services. We investigated the factors associated with uptake of antiretroviral treatment through a primary healthcare system in rural South Africa.

**Methods:**

Detailed demographic, HIV surveillance and geographic information system (GIS) data were used to estimate the proportion of HIV positive adults accessing antiretroviral treatment within northern KwaZulu-Natal, South Africa in the period from initiation of antiretroviral roll-out until the end of 2008. Demographic, spatial and socioeconomic factors influencing the likelihood of individuals accessing antiretroviral treatment were explored using multivariable analysis.

**Results:**

Mean uptake of ART among HIV positive resident adults was 21.0% (95%CI 20.1-21.9). Uptake among HIV positive men (19.2%) was slightly lower than women (21.8%, P = 0.011). An individual's likelihood of accessing ART was not associated with level of education, household assets or urban/rural locale. ART uptake was strongly negatively associated with distance from the nearest primary healthcare facility (aOR = 0.728 per square-root transformed km, 95%CI 0.658-0.963, *P *= 0.002).

**Conclusions:**

Despite concerns about the equitable nature of antiretroviral treatment rollout, we find very few differences in ART uptake across a range of socio-demographic variables in a rural South African population. However, even when socio-demographic factors were taken into account, individuals living further away from primary healthcare clinics were still significantly less likely to be accessing ART

## Background

Recent years have seen substantial progress being made in the roll-out of antiretroviral therapy (ART) to populations in sub-Saharan Africa [1]. Many challenges remain in achieving access to antiretroviral treatment for all those in need, particularly in more rural parts of sub-Saharan Africa where often there is weak, if any, public health infrastructure. In areas with hyperendemic HIV infection, delivery of ART is seen as an important component of multi-faceted prevention measures [2] and increasingly attention is focused on whether antiretrovirals could be targeted more widely to have a direct impact on population HIV transmission [3]. Such a strategy, if implemented, would require substantially higher levels of antiretroviral treatment coverage than current targets and a detailed understanding of the extent to which current systems are able to deliver ART is increasingly important.

South Africa carries the world's greatest burden of HIV infection, with estimates that it is home to approximately 17% of the world's HIV positive population [4]. Worst affected within South Africa is the province of KwaZulu-Natal, home to approximately 1.5 million HIV positive individuals and where HIV prevalence is greater than 50% in some age groups [5]. The province is mostly rural [6] and despite a decentralized primary healthcare system, many patients have difficulty travelling to their nearest healthcare facility [7]. The challenges posed by ART delivery in the region were the subject of early modeling exercises prior to antiretroviral roll-out, with a particular focus on equity of ART delivery [6,8,9]. The realities of roll-out have been more varied than those models originally envisaged and ART is not routinely available in many primary care facilities.

Here we describe the evolution of antiretroviral treatment through a primary healthcare service in a rural South African setting where at a sub-district level there exists substantial geographical heterogeneity in HIV prevalence [10]. We use detailed demographic, HIV surveillance and geographical information systems (GIS) data to estimate the proportion of the population accessing ART and explore geographical variation in ART uptake across the study area. In addition, we investigate whether socioeconomic and geographic factors are associated with the likelihood of ART uptake.

## Methods

The study was carried out in the Hlabisa sub-district in Umkhanyakude district, northern KwaZulu-Natal. The district is the third most deprived in South Africa [11]. Since 1999, the Africa Centre for Health and Population Studies http://www.africacentre.ac.za has carried out established a demographic surveillance area (DSA) within a portion of this sub-district. The DSA has a population of approximately 87,000 within an area of 438 km^2 ^including deep rural areas, a township and peri-urban informal settlements. At any point in time, one-third of the population under surveillance, who although members of households in the area, do not physically reside in the surveillance area [11].

Since the beginning of 2003, HIV infection status of adults has been determined through a separate annual sero-surveillance [12]. HIV prevalence in this population has steadily increased since the early 1990s [13,14] to 21·5% in 2004. Overall, 27% of female and 13·5% of male residents were HIV-infected in 2004, HIV prevalence was highest in the five-year age groups of 25-29 years in women (51%) and 30-34 years in men (44%)[15]. The geographical distribution of HIV infection is not uniform and ranges from <10% in some of the more rural parts of the surveillance area to ≥35% in some of the high density settlements located along the National Road[10].

### Delivery of care and antiretroviral treatment

The Hlabisa HIV treatment and care program has been described in detail elsewhere[16]. Briefly, the program covers the whole sub-district of Hlabisa, an area with a population of approximately 228,000 people. Six of the clinics in the sub-district lie within the demographic surveillance area described above; the DSA covers approximately 40% of the population in the sub-district and is the focus of this analysis. Since 2004, ART has been provided free of charge through government clinics in the area. Public sector provision has undergone rapid expansion [17], aided in the area of study by NGO support. The service began in late 2004 based at the district hospital (Phase 1). In August 2005, provision was added to a community health centre in the township within the area of surveillance and all primary healthcare clinics began monitoring CD4 counts and providing antiretroviral treatment (Phase 2). By December 2006, 14 primary healthcare facilities had clinician support for the care and treatment of patients requiring ART (Phase 3).

Patients were eligible for ART based on standardised national guidelines [18]. Prior to initiation of antiretroviral treatment patients were monitored with periodic CD4 counts. First line ART was a standardised combination of two nucleoside reverse transcriptase inhibitors (NRTIs; stavudine and lamivudine) and one non-nucleoside reverse transcriptase inhibitors (NNRTIs; efavirenz or nevirapine). Following ART initiation, patients were reviewed monthly by a counsellor and are offered CD4 counts and viral load testing on a 6 monthly basis.

### Estimation of numbers of adults receiving ART by catchment area

The demographic surveillance area was divided into six clinic catchments using a validated GIS model of travel time to clinic described previously [19]. Individuals age 15 or older initiating HAART in the sub-district (or transferring in their care) between program inception in August 2004 and 31^st ^December 2008 were eligible for inclusion in the study. Individuals were excluded if they were lost to follow-up (did not attend for three consecutive appointments), died or were transferred out of the program. The catchments provide an intuitive means of dividing up the study area into sub-areas and did not exclude patients living in these areas who were getting their ART elsewhere in the sub-district. To allow comparison of where patients actually receive care with where they would be expected to receive care based on physical proximity to clinics, patients were assigned directly to a clinic catchment in the surveillance area using the GIS location of their homestead of residence (obtained through direct linkage to a demographic database). Strict matching criteria were applied by dedicated data handling staff which meant a person was only linked either by their unique South African identification number, or if both first and surnames matched. The remaining patients (who reportedly lived in the surveillance area but could not be directly linked) were assigned to clinic catchments using the local area (neighbourhood or izigodi) information provided by the patient. In isolated cases where the local area spanned multiple clinic catchments, patients were stochastically assigned to one of the overlapping catchments according to the degree of overlap between the areas. Ethical permission was received from the local Department of Health and University of KwaZulu Natal (E134/06) and verbal consent was obtained from individuals participating in demographic surveillance. Individual written informed consent was given for participation in HIV surveillance. Because the demographic surveillance collects information on both residents and non-residents of households in the surveillance area [11], we quantified the proportion of ART patients (among linked patients) who were not normally resident in the surveillance area but who returned periodically to visit their families and to receive ART.

The underlying numbers of individuals in the population with HIV were estimated from surveillance data. Because the population consenting to an HIV test differs from the total population for testing, we produced age-sex standardised (to the eligible population in each catchment area) estimates of the total number of HIV positive resident adults in each clinic catchment. We then used to this information to estimate the proportion of ART uptake by clinic catchment (number of residents adults receiving ART/number of resident adults with HIV infection).

### Analysing the factors associated with ART uptake

To assess antiretroviral treatment uptake across socio-economic criteria, we used individuals in the population-based 2008 cohort to compare HIV positive residents (≥15 years of age) on antiretroviral treatment (N = 1,251) with HIV positive residents identified by the population-based surveillance who were not recorded as receiving ART (N = 1,033) using a binary logistic regression (weighted according to the characteristics of the underlying HIV positive population under surveillance). In addition to age and sex, co-variates studied were years of education, household asset index, urban/rural/peri-urban locale and Euclidean distance to the nearest ART clinic (square-root transformed km). Household asset index was calculated using previously described principal components analysis [15]. The 2007 household socio-economic variables used in the analysis are collected routinely by the surveillance system using methodology described previously [11]. Statistical and spatial analysis were performed using STATA v 10 (StataCorp, USA) and Mapinfo 10.0 (Rockware, USA).

## Results

The total number of patients ever initiated on ART in the program increased to 7,576 by the end of 2008 with 6,354 patients actively on ART (67% were female). Of those actively receiving ART, 2,412 were attending one of the six pre-defined clinic catchments (see Table 1). An estimated 1660 (69%) of these were resident in the six catchment areas whilst the remainder were migrants who ordinarily lived elsewhere but returned to the area frequently. Seventy four percent of patients within the DSA were matched directly to clinic catchment areas, twenty six percent allocated as described in methods. The median distance travelled by a patient in the program living in the study area to access ART fell from 34.2 km when treatment was only available at the district hospital, to 8.5 km when treatment was available through the community healthcare facility, to 3.1 km when treatment was available through all primary healthcare clinics. By the end of 2008, 70% of patients received treatment from their nearest clinic (defined on the basis of estimated travel time[20]).

**Table 1:** Characteristics of the six catchment areas studied

	Catchment Population	Total patients actively on ART	Estimated number of residents receiving ART	HIV Prevalence Residents (%)	Estimated Residents HIV+	Estimated percentage (95% CI) residents on ART
**1**	Rural	90	62	17.3	250	24.7 (19.9 - 29.5)
**2**	Rural	187	129	15.1	463	27.7 (24.1 - 31.3)
**3**	Urban/peri-urban	1063	732	26.7	3372	21.6 (20.4 to 22.9)
**4**	Rural	171	118	19.3	504	23.2 (20.0 - 26.6)
**5**	Peri-urban	488	336	23.1	1831	18.3 (16.7 - 19.9)
**6**	Peri-urban/rural	413	284	18.3	1475	19.2 (17.4 - 21.0)

**Total**		**2412**	**1660**	22.1	**7895**	**21.0 (20.2 - 21.8)**

**Figure 1:**
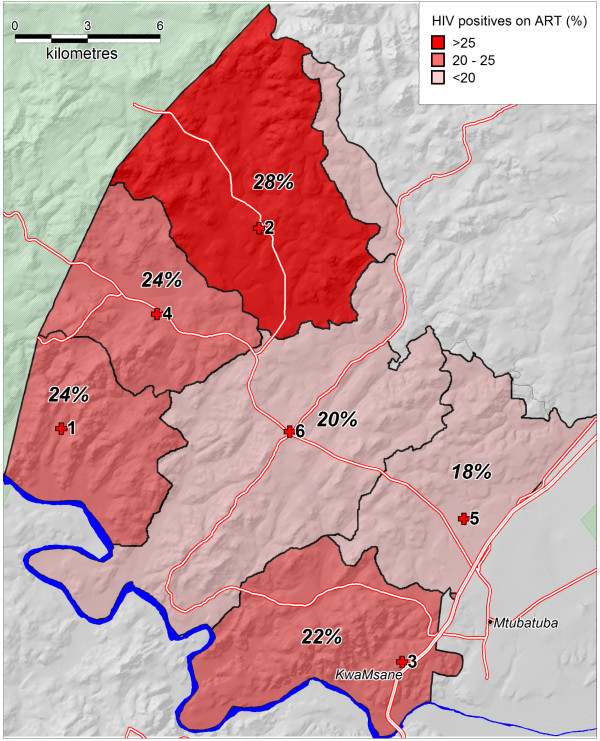
**Mean uptake of antiretroviral treatment (%) by HIV positive adults in adjacent clinic catchment areas**.

The mean population uptake of ART among HIV positive residents was 21.0% (95% C.I. = 20.1 - 21.9) and ART uptake in different clinic catchment areas ranged from 18.3 to 27.7% (Figure 1). The highest uptake was found in one of the more rural catchment areas. Overall, antiretroviral treatment uptake was slightly higher in HIV-positive women (21.8%, 95% CI = 20.7 - 22.9%) by comparison to HIV positive men (19.2%, 95 CI = 17.7 - 20.8%, p-value of difference = 0.011). Uptake was lowest in the youngest age-groups where the majority of HIV infections are recently acquired. In this population, uptake of ART in males lagged behind females probably reflecting the later age at infection in males - it only begins to approach parity in the 40-45 year age group. ART uptake was highest among HIV positive females in the 50-54 year age group (45%) and thereafter declined to 20% in the ≥60 age group. Males showed a similar pattern with 36% of 50-54 year old HIV positive males on ART. The seemingly relatively high coverage of ART in the very young and old age groups in HIV positive males likely reflects the instability in these estimates due to small numbers of patients on treatment.

The characteristics of individuals in the analysis comparing linked HIV positive patients on ART against infected individuals not on ART is given (Table 2). An multi-variable analysis exploring factors influencing an individual's likelihood of accessing ART (comparing the linked HIV positive patients on ART against infected individuals not on ART) showed that HIV positive men were less likely to access antiretroviral treatment in comparison to women, but the difference was not significant (Table 3). As would be expected on the basis of ART need, age was a highly significant predictor of uptake - the median was 30.5 year for the untreated group and 36.3 for the group on ART. The adjusted odds of accessing ART remained high throughout the ages 30-54 and peaked in the 50-54 year age group.

**Table 2:** Characteristics of individuals included in analysis of determinants of ART uptake (N = 2,284)

	Not on ART (%)	95% CI	On ART (%)	95% CI	Pearson Chi2
**Age Group**					
15-19 (n = 143)	88.8	[79.4,94.2]	11.2	[5.8,20.6]	
20-24 (n = 312)	79.5	[74.8,83.5]	20.5	[16.5,25.2]	
25-29 (n = 382)	59.2	[51.8,66.2]	40.8	[33.8,48.2]	
30-34 (n = 385)	43.6	[37.8,49.7]	56.4	[50.3,62.2]	
35-40 (n = 330)	39.7	[33.3,46.4]	60.3	[53.6,66.7]	
40-45 (n = 240)	43.3	[38.1,48.7]	56.7	[51.3,61.9]	
45-50 (n = 194)	50.5	[44.5,56.5]	49.5	[43.5,55.5]	
50-54 (n = 155)	41.9	[33.6,50.8]	58.1	[49.2,66.4]	
55-60 (n = 73)	54.8	[48.6,60.8]	45.2	[39.2,51.4]	
> 60 (n = 70)	62.9	[52.2,72.4]	37.1	[27.6,47.8]	**< 0.001**
					
**Sex**					
FEM (n = 1,757)	56	[48.4,63.3]	44	[36.7,51.6]	
MAL (n = 527)	50.7	[41.5,59.8]	49.3	[40.2,58.5]	**0.031**
					
**Years of Education Attained**					
0 (n = 334)	52.4	[46.1,58.6]	47.6	[41.4,53.9]	
1 (n = 25)	48	[30.3,66.2]	52	[33.8,69.7]	
2 (n = 46)	56.5	[40.1,71.7]	43.5	[28.3,59.9]	
3 (n = 93)	52.7	[42.6,62.5]	47.3	[37.5,57.4]	
4 (n = 89)	49.4	[39.7,59.2]	50.6	[40.8,60.3]	
5 (n = 89)	47.2	[38.1,56.5]	52.8	[43.5,61.9]	
6 (n = 94)	47.9	[36.6,59.3]	52.1	[40.7,63.4]	
7 (n = 177)	57.1	[48.1,65.6]	42.9	[34.4,51.9]	
8 (n = 182)	59.9	[46.8,71.7]	40.1	[28.3,53.2]	
9 (n = 173)	62.4	[47.2,75.6]	37.6	[24.4,52.8]	
10 (n = 290)	57.6	[48.4,66.3]	42.4	[33.7,51.6]	
11 (n = 238)	60.5	[47.6,72.1]	39.5	[27.9,52.4]	
12 (n = 454)	50.4	[44.0,56.9]	49.6	[43.1,56.0]	**0.062**
					
**Assets Index (1 = poorest)**					
1 (n = 403)	54.8	[47.5,62.0]	45.2	[38.0,52.5]	
2 (n = 440)	56.1	[47.9,64.0]	43.9	[36.0,52.1]	
3 (n = 495)	56	[47.8,63.8]	44	[36.2,52.2]	
4 (n = 503)	55.5	[46.8,63.8]	44.5	[36.2,53.2]	
5 (n = 381)	51.2	[43.5,58.8]	48.8	[41.2,56.5]	
Missing (n = 62)	51.6	[35.0,67.8]	48.4	[32.2,65.0]	0.709
					
**Urban/Rural**					
Peri-Urban (n = 822)	51.5	[41.6,61.2]	48.5	[38.8,58.4]	
Rural (n = 1,296)	58.2	[49.7,66.2]	41.8	[33.8,50.3]	
Urban (n = 166)	44.6	[35.1,54.5]	55.4	[45.5,64.9]	< 0.001
					
**Distance to Clinic**					
< 1 Km (n = 306)	45.4	[37.9,53.2]	54.6	[46.8,62.1]	
1-2 Km (n = 597)	52.8	[45.0,60.4]	47.2	[39.6,55.0]	
2-3 Km (n = 457)	56.5	[49.0,63.6]	43.5	[36.4,51.0]	
3-4 Km (n = 378)	52.6	[45.0,60.2]	47.4	[39.8,55.0]	
4-5 Km (n = 228)	61.8	[52.8,70.2]	38.2	[29.8,47.2]	
> 5 Km (n = 318)	62.6	[52.4,71.8]	37.4	[28.2,47.6]	< 0.001
					
**Total (n = 2,284)**	**54.8**	**[48.5,60.9]**	**45.2**	**[39.1,51.5]**	

**Table 3:** Results of a logistic regression comparing the characteristics of HIV positive residents (≥15 years of age) on treatment (N = 1,251) with HIV positive residents who were not recorded as receiving treatment (N = 1,033).

	Participants(n = 2,284)				
Covariate	Unadjusted OR*	P≥|z|	Adjusted OR*	P≥|z|	95% CI
**Sex**					
F*	1		1		
M	1.098	0.368	0.875	0.216	(0.708 to 1.081)
**Age**					
15-19*	1		1		
20-24	1.947	0.030	2.001	0.025	(1.092 to 3.669)
25-29	4.904	< 0.001	4.998	< 0.001	(2.820 to 8.859)
30-34	10.499	< 0.001	11.065	< 0.001	(6.230 to 19.653)
35-40	11.536	< 0.001	12.525	< 0.001	(6.949 to 22.574)
40-45	9.610	< 0.001	10.579	< 0.001	(5.742 to 19.494)
45-50	7.944	< 0.001	9.297	< 0.001	(4.960 to 17.426)
50-54	11.480	< 0.001	14.020	< 0.001	(7.309 to 26.890)
55-60	5.564	< 0.001	6.726	< 0.001	(3.169 to 14.276)
≥60	4.632	< 0.001	5.834	< 0.001	(2.737 to 12.437)
**Years Of Education**					
per unit	0.987	0.213	1.022	0.128	(0.995 to 1.063)
**Assets Index**					
1 (poorest)*	1		1		
2	0.954	0.748	0.932	0.649	(0.688 to 1.262)
3	0.921	0.564	0.842	0.258	(0.624 to 1.135)
4	0.983	0.904	0.829	0.237	(0.607 to 1.131)
5 (wealthiest)	1.115	0.475	0.984	0.927	(0.702 to 1.379)
(Missing)	1.035	0.906	0.684	0.211	(0.377 to 1.241)
**Urban/Rural Status**					
Peri-urban	0.883	0.498	1.042	0.838	(0.699 to 1.554)
Rural	0.654	0.017	0.941	0.768	(0.628 to 1.410)
Urban*	1		1		
**SqrtKmToNearestClinic**					
per unit	0.671	< 0.001	0.728	0.002	(0.618 to 0.990)

* Weighted according to the characteristics of the underlying HIV positive population

**Figure 2:**
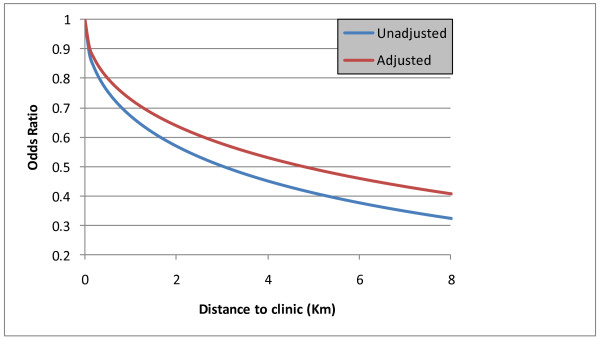
**Overall likelihood of accessing antiretroviral treatment with distance from nearest primary healthcare facility (km)**.

**Figure 3:**
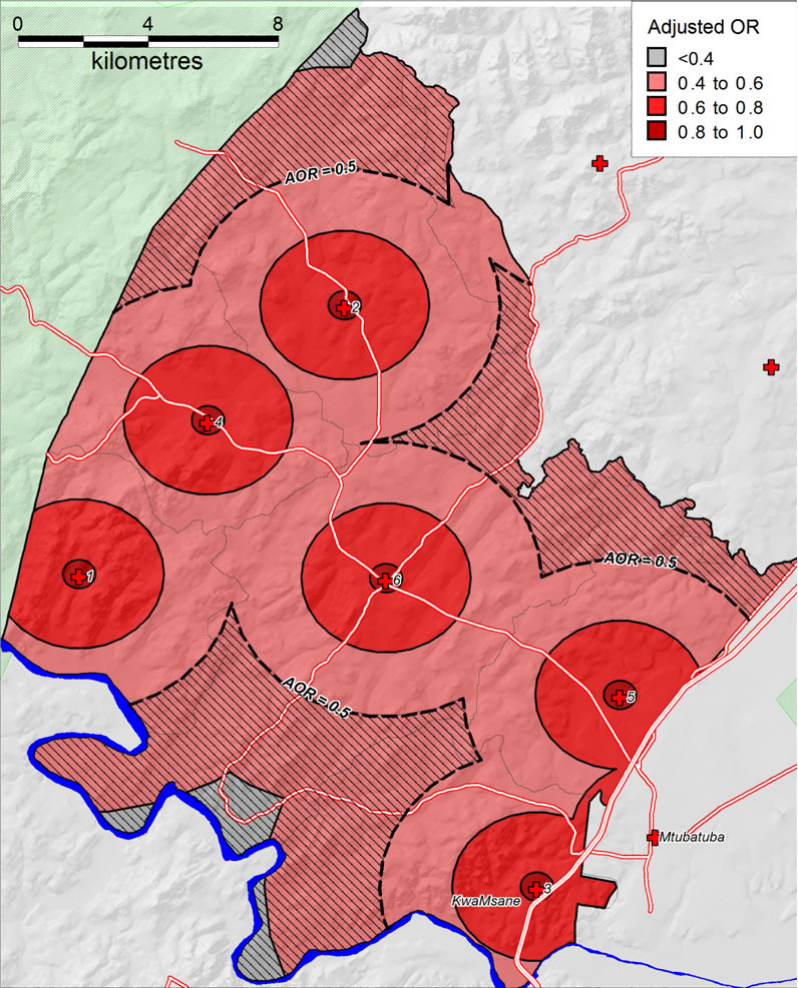
**Likelihood (OR) for HIV positive adults accessing ART as a function of distance from nearest primary healthcare facility**.

Only rural/urban locale was a significant predictor of uptake in univariate analysis but in multivariable analysis the key predictor of ART uptake among the infected population was distance to the nearest health facility. ART uptake amongst HIV positive individuals within 1 km of a clinic was estimated to be 25.9% (95% CI; 23.1-28.8). The likelihood of accessing ART decreased by 27% with every square-root transformed km to nearest health facility. This equates to an initial steep decrease in likelihood of accessing ART with increasing distance (Figure 2) but the rate of decrease attenuates as distance increases. At just 4.78 km (unadjusted = 3.04 km) from the nearest clinic the odds of an HIV positive individual accessing ART are half those of an infected person living next door to a health facility holding all other factors constant (Figure 3). Approximately 31% of the study area (containing 19% of the population) is further than 4.78 km away from the nearest health facility.

Educational attainment, household wealth quintile and urban/rural locale did not significantly affect the likelihood of an HIV positive individual receiving ART in the multi-variate analysis (Table 3). In addition, we found no relationship between school attainment and uptake among infected individuals.

## Discussion

This represents one of the largest studies to date investigating factors related to antiretroviral uptake in an African setting. Through linkage of the population-based HIV surveillance data to the Hlabisa treatment and care program [16] we have been able to examine in detail the factors associated with ART uptake in a rural area. Despite questions raised [21,8,22] around the ability of ART programs in rural settings to reach vulnerable populations, we find remarkably little socio-demographic difference between the HIV positive populations accessing/not accessing ART. We do, however, find that physical distance from primary health clinics is a significant obstacle to accessing ART even when other socio-demographic factors are taken into account. Our data estimate ART uptake within 1 km of a clinic to be 25.9% (compared to 21.0% overall), falling rapidly with increasing distance from a clinic. This is despite an accelerated attempt towards decentralization of services in the area in question. Put more starkly, at distances of only 5 km from the nearest clinic, the odds of an HIV positive individual accessing ART are less than half those of an infected person living immediately next to a health facility holding all other factors constant. Contrary to expectation, no effect was seen due to level of education or measures of household wealth.

In the region in question, the provision of ART through primary healthcare facilities reduced the median distance that patients travel to access ART from 34.2 km to 3.1 km. This reduction in travel distances stands in comparison to estimates of 20 km or upwards used in early modelling of resource allocation [9]. To achieve more uniform levels of ART uptake, consideration will have to be given to the cost-effectiveness of alternative ART delivery strategies (including, but not limited to, mobile treatment centres and more comprehensive home support services). Our results suggest that by making ART services more physically accessible, a relative increase in population ART uptake approaching 20% could be achieved.

The study area differs from many others in having rapidly decentralised ART delivery to primary care. We find that through this system it was possible to deliver ART to approximately 21% of the total HIV positive adult population over limited period of time within a relatively poor, rural area. This proportion accessing ART exceeds the target of 15% widely used in estimates of ART need at the beginning of roll-out [23] (and could be termed >100% coverage). However, such targets are dynamic in areas where antiretroviral roll-out is maturing and new infection rates remain high [24], and can differ between regions [25] and for these reasons we focus here on the proportion of infected individuals accessing ART. Recently we have started to model the epidemic in this area using STDSIM [26]. Initial estimates indicate that at the end of 2008, the 21% uptake of ART among the HIV positive population translates into a coverage figure of approximately 66% (Jan Hontelez, pers comm).

As well as the overall proportion of a population accessing ART, there are different notions of ethical treatment delivery. One principle highlighted in previous work is egalitarian equity (distribution of healthcare equally among groups that differ in socioeconomic circumstances) [8,22,27]. The observation that the profile of household assets for those accessing ART does not differ significantly from that of the population infected with HIV, suggests that the ethical principle of egalitarian equity is being observed with regard to wealth. Locally, individuals in the poorest households are no less likely to utilize ART than those in wealthier ones (either univariately or holding all other factors constant). Contrary to recent reports from other settings suggesting large gender disparities in access to HIV treatment and care [28,29], we show that HIV positive men are only slightly less likely to have accessed ART in comparison to women. However, this is not to say that significant sex differences do not exist, for example local data suggests that amongst individuals not yet eligible for HAART, retention rates within the program are poorer for men [30] and that men are more likely to access ART programs with evidence of advanced disease [17]. The significant differences in age observed are expected and a consequence of the time delay between HIV infection and progression to the point of ART eligibility which studies find to be quite consistent across sub-Saharan Africa [31].

The speed with which services can be scaled-up has to be balanced against the quality of care it is possible to deliver and ultimately, the most important outcomes of widespread ART delivery will be the impact on population mortality and ongoing HIV transmission. Estimates from the study area suggest an important early impact on population mortality, but that HIV remains the leading cause of death [32], and HIV incidence remains high [5]. Local data suggests that in this early phase of ART delivery at least, outcomes for ART showed no evidence of decline [17] and are broadly similar to those described elsewhere [33], though such data is difficult to compare between sites [34]. The development of services in the area of study was support by local NGOs with PEPFAR funding which is not the case in many other settings. Whether such outcomes can be maintained as services reach capacity is an important consideration, particularly as discussions begin around the possibility of reaching far higher levels of ART uptake, both in the implementation of new WHO treatment guidelines and as a means to decrease rates of HIV transmission [3].

There are some limitations to the work presented here; the imperfect linkage between ART program and HIV surveillance data translates into relatively small false negative ratio of 6.5% (those on ART but not designated as such) in the individual-based risk factor analysis. This could result in a slight ascertainment bias of the results towards the null hypothesis. However, this could not create a spurious positive finding and would be unlikely to impact on any of the "null" findings (sex, education, household wealth, urban/rural locale) as none of these predictors bordered on statistical significance. We were not able to measure the small numbers of individuals accessing ART through non-governmental sources which might include family practitioners or other care providers in the area. During the period in question, the proportion of individuals receiving care in these settings was less than 5% of the total number. These factors mean that estimates of ART uptake provided here should be taken to refer to public sector delivery, the major ART source in this area. They are likely to be a lower estimate for the total population uptake of antiretroviral treatment overall, but that the underestimate is likely to be small. Such methodological issues will be important when comparing data from different settings and one interesting and unexpected finding of this study has been the large proportion (31%) of individuals receiving ART in the sub-district who are not normally resident in the area (migrants) but return frequently to be with their families and receive their ART. Failure to account for this could lead to inflated uptake estimates.

Strategies considering the wider use of antiretrovirals for preventing HIV transmission will require not only much higher levels of treatment uptake, but also high levels of VCT uptake. We have not attempted to address the overall uptake of VCT testing and knowledge of HIV status as individual data is not collected locally within government services. However, some minimum estimate of the numbers of HIV positive aware of their status can be derived from the number of different individuals accessing care (as determined by unique individuals attending for CD4 testing) before eligibility for ART compared to those receiving ART. Locally that ratio is approximately 1:1 suggesting a minimum bound for the proportion of the HIV positive population accessing care of at least 40%. The true level of VCT uptake is likely to be higher, to some extent for the reasons described above in relation to estimates of treatment uptake, but also because a significant number of individuals are probably lost from services prior to CD4 testing following a positive VCT result.

## Conclusions

Taken overall this work demonstrates the feasibility of delivering antiretroviral treatment to a significant proportion of the HIV positive population through a decentralised primary care system. Through this system it was possible to achieve equitable ART delivery, at least for measures based on household wealth and educational attainment. However, despite this process of decentralisation, physical access to ART remains a challenge to be overcome.

## Competing interests

The authors declare that they have no competing interests.

## Authors' contributions

GC, FT, TB and MLN contributed to the study design, analysis and writing of the paper. All authors have read an approved the final manuscript.

## Pre-publication history

The pre-publication history for this paper can be accessed here:

http://www.biomedcentral.com/1471-2458/10/585/prepub
